# Histogram analysis with automated extraction of brain-tissue region from whole-brain CT images

**DOI:** 10.1186/s40064-015-1587-1

**Published:** 2015-12-18

**Authors:** Masatoshi Kondo, Koji Yamashita, Takashi Yoshiura, Akio Hiwatash, Takashi Shirasaka, Hisao Arimura, Yasuhiko Nakamura, Hiroshi Honda

**Affiliations:** Department of Medical Technology, Kyushu University Hospital, 3-1-1 Maidashi, Higashi-ku, Fukuoka 812-8582 Japan; Graduate School of Health Sciences, Kumamoto University, 4-24-1 Kuhonji, Kumamoto, 862-0976 Japan; Department of Clinical Radiology, Graduate School of Medical Sciences, Kyushu University, 3-1-1 Maidashi, Higashi-ku, Fukuoka 812-8582 Japan; Department of Radiology, Graduate School of Medical and Dental Sciences, Kagoshima University, 8-35-1 Sakuragaoka, Kagoshima, 890-8544 Japan; Department of Radiological Technology, Kumamoto University Hospital, 1-1-1 Honjyo, Kumamoto, 860-8556 Japan

**Keywords:** Whole-brain CT, Brain-tissue region, Automated extraction, Histogram analysis

## Abstract

To determine whether an automated extraction of the brain-tissue region from CT images is useful for the histogram analysis of the brain-tissue region was studied. We used the CT images of 11 patients. We developed an automatic brain-tissue extraction algorithm. We evaluated the similarity index of this automated extraction method relative to manual extraction, and we compared the mean CT number of all extracted pixels and the kurtosis and skewness of the distribution of CT numbers of all extracted pixels from the automated and manual extractions. The similarity index was 0.93. The mean CT number and the kurtosis and skewness from the automated extraction were 35.0 Hounsfield units, 0.63, and 0.51, respectively, and were equivalent to those from the manual extraction (35.4 Hounsfield units, 0.59, and 0.46, respectively). The automated extraction of the brain-tissue region from whole-brain CT images was useful for histogram analysis of the brain-tissue region.

## Background

Brain CT is the first-choice imaging modality in the diagnosis of many intracranial diseases. Previous studies have demonstrated that quantitative region-of-interest analysis of brain CT images provides useful evidence in the assessment of early ischemic stroke, early postmortem change, and prognosis in resuscitated patients (Dzialowski et al. 2004; Kim et al. 2004; Sugimori et al. 2012; Takahashi et al. 2010). However, regional analyses may include a component of subjective evaluation, and we propose that whole-brain analysis would be a more useful and objective method of identifying brain damage.

Several semi-automated brain segmentation methods have been proposed for extracting region of brain tissue from CT images (Maksimovic et al. 2009; DeLeo et al. 1985; Soltanian-Zadeh and Windham 1997). However, these methods involve user-dependent procedures, and automated methods are more reproducible (Admiraal-Behloul et al. 2005) and consistent (Carmichael et al. 2005) than manual or semi-automated methods. (Hu et al. 2005) applied an automated extraction method that applied fuzzy c-means clustering to brain CT images from a single subject, and (Ganesan and Radhakrishnan 2009) used an automated method that applied a genetic algorithm to contrast-enhanced brain CT images. (Gupta et al. 2010) reported on an automated method that incorporated analysis of intensity histograms; however, as it is generally assumed that the cerebrospinal-fluid ventricle is the largest connected component within the brain, this method might result in extraction errors in subjects who have diffuse brain damage that involves narrowing or effacement of the ventricle (Yanagawa et al. 2005; Cunningham and Twickler 2000).

CT histogram analyses have been reported to provide added diagnostic value in several fields (Gould et al. 1991; Bae et al. 2003; Chaudhry et al. 2012; Sakai et al. 1994). To the best of our knowledge, the histogram analysis of a brain-tissue region from brain CT images has not been reported previously. Since, there is no commercially available software to extract the brain-tissue region from whole brain unenhanced CT images, first we propose a simple automated method for extracting the brain-tissue region from brain CT images, then we evaluated it’s performance compared to manual extraction in multiple subjects.

## Methods

### Whole-brain CT image

This retrospective study was approved by our institution’s review board. We retrospectively analyzed the CT images of 11 patients who had mild head injuries without loss of consciousness, and who underwent whole-brain unenhanced CT examinations in our hospital between January and March 2009. The age range of the subjects was 16–40 years (mean age, 27.1 years) and there were seven males and four females. Two subjects were diagnosed with subcutaneous hematoma, and the other nine cases were unremarkable. Table 1 slides the scanning parameters used. Images were reconstructed by the scanner with use of the following parameters: slice thickness 4.0 mm, reconstruction kernel, head, filtered back projection, image matrix size 512 × 512 pixels, display field of view, 24.0 cm.

**Table 1:** Scanning parameters for the unenhanced whole-brain CT images obtained from 11 patients with mild head injuries

Parameter	Value
Collimation (mm)	4 × 2
Tube voltage (kV)	120
Tube amplitude (mA)	250
Rotation speed (s/rot)	1.5
Slice thickness (mm)	4
CT dose index (mGy)	89.2

### Automated extraction of brain tissue

**Fig. 1:**
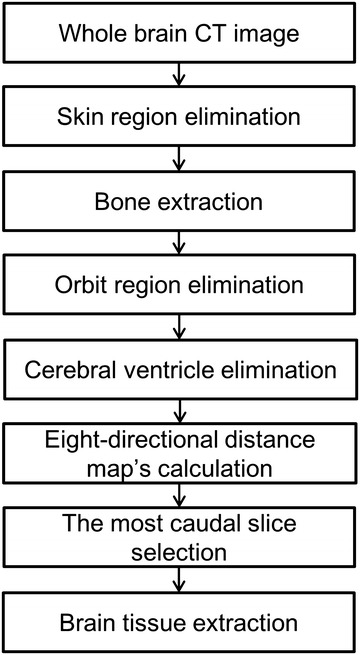
Flowchart of the method used for automatically extracting the brain-tissue region from whole-brain CT images

**Fig. 2:**
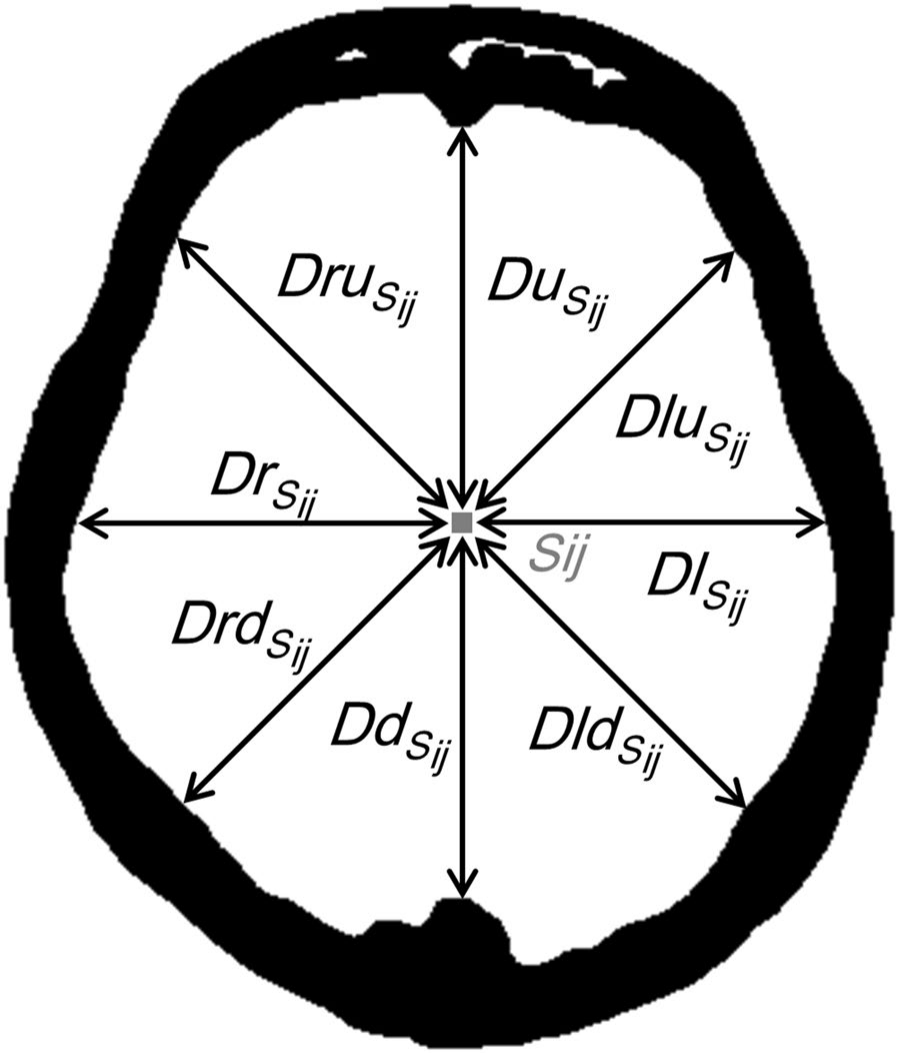
The eight directions (*bidirectional arrows*) from sample pixel (*S*_*ij*_) to the inner boundary of the skull used for calculating *Dmap*

The brain-tissue region was extracted in contiguous slices from the vertex to the foramen magnum. The extraction algorithm was based on a seven-step process (Fig. 1). First, because the density of the skin region is high, the skin region was eliminated to allow the skull region to be segmented correctly in the subsequent step. The area that remained after subtraction of the *binarized area* from the *non*-*erosion binarized area* after seven consecutive erosions with use of a 3 × 3 2D kernel with binarization at a threshold of −200 Hounsfield units (HU) was defined as the skin region, and eliminated. Second, the skull region was extracted with use of a threshold of >84 HU (Hara et al. 2007). Third, binary dilations, with use of a 3 × 3 2D kernel for closing the skull region, were performed five times to eliminate the orbital region. Fourth, the cerebral ventricle was eliminated by use of a threshold of <22 HU (Gawler et al. 1976). Fifth, the distance between each pixel (*S*_*ij*_) and the skull was measured in eight directions (Fig. 2) and used for calculation of *Dmap*_*Sij*_ according to Eq. 1

1 $$Dmap_{Sij} = Du_{sij} + Dlu_{sij} + Dl_{sij} + Dld_{sij} + Dd_{sij} + Drd_{sij} + Dr_{sij} + Dru_{sij},$$

where each *D* is the shortest distance from the *S*_*ij*_ to the inner boundary of the skull in each of the eight directions (up, left-up, left, left-down, down, right-down, right, and right-up, respectively) (Fig. 2). When all *D* values are not measureable, we define the *Dmap* is 0. Sixth, matching was performed with a 2D 20-pixel-diameter circular template on the *Dmap* between 100 and 250 pixels for detection of the foramen magnum. The foramen magnum was considered to be located in the slice at which the circle template matched >100 pixels, and this was used as the most inferior slice in the analysis. Finally, pixels with *Dmap* > 0 from the most inferior slice to the most superior slice were extracted as brain tissue.

### Manual extraction of brain-tissue region

A radiologic technologist with 5 years’ experience manually extracted the brain-tissue region by using the image editor tool Paint (Microsoft Corp., Redmond, WA) and the “adjust-color threshold” function of ImageJ (NIH Image, Bethesda, MD). The pixels of the ventricles, the cisterns, the blood vessels, and the cranial nerves were carefully excluded. The extractions were confirmed by a neuroradiologist with 13 years’ experience.

### Accuracy analysis of automated extraction

First, the number of pixels extracted by the automated procedure was compared to the number of pixels extracted by the manual procedure (Atlins and Machiewich 1998). The similarity index (Eq. 2) and rates of false positives (Eq. 3) and false negatives (Eq. 4) were calculated for comparison of the two procedures:

2 $${\text{Similarity}}\,{\text{index}}\, = \,2\, \times \,{{\left( {Aauto \cap Amanual} \right)} \mathord{\left/ {\vphantom {{\left( {Aauto \cap Amanual} \right)} {\left( {Aauto\,{ + }\,Amanual} \right)}}} \right. \kern-0pt} {\left( {Aauto\,{ + }\,Amanual} \right)}}$$

3 $${\text{False}}\,{\text{positive}} = \,100 \times {{\left( {Aauto \cap \overline{Amanual} } \right)} \mathord{\left/ {\vphantom {{\left( {Aauto \cap \overline{Amanual} } \right)} {Amanual}}} \right. \kern-0pt} {Amanual}}$$

4 $${\text{False}}\,{\text{negative}} = \,100 \times {{\left( {\overline{Aauto} \cap Amanual} \right)} \mathord{\left/ {\vphantom {{\left( {\overline{Aauto} \cap Amanual} \right)} {Amanual}}} \right. \kern-0pt} {Amanual}}$$

where *Aauto* is the number of pixels extracted automatically, and *Amanual* is the number of pixels extracted manually.

### Histogram analysis

The frequency distribution of the CT numbers from 22 to 84 HU was calculated for each extraction method, and the mean CT number of this histogram was quantified. For quantification of the kurtosis and skewness, the histogram was limited to 22–50 HU to avoid the influence of expected heavy tail distributions.

### Statistical analysis

The number of extracted pixels, the mean CT number of the extracted pixels, and the kurtosis and skewness of the histogram were compared across the automated and manual extraction methods by use of two-tailed Student’s t-tests for paired samples. Data are expressed as mean ± SD. The relationship between the mean CT number, kurtosis, and skewness of the automated and manual extraction methods was evaluated by use of Pearson’s correlation. All statistical analyses were performed with commercially available software (JMP, version 9.0, SAS Institute, Cary, NC), and a *P* value < 0.05 was considered to indicate significance.

## Results

### Extraction time

The automated extraction of the brain-tissue region was implemented on Cygwin (www.cygwin.com). For one database of 40 slices, the analysis time for the automated extraction was 33 ± 2 s with a 2.8-GHz CPU with 8 GB RAM. The analysis time for manual extraction of the same database was 4.2 ± 0.4 h.

### Accuracy of automated extraction of brain-tissue region

**Fig. 3:**
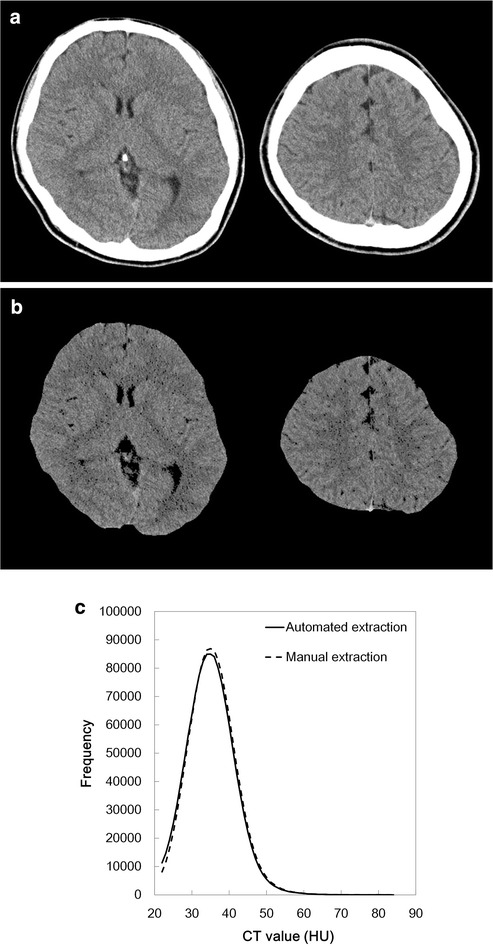
Representative CT images (**a**) extracted results from the corresponding slices (**b)**, and CT histogram (**c**; *solid line*: automated extraction; *dashed line*: manual extraction) from a 21-year-old male. The similarity index was 0.941, which was the highest of all the databases

**Fig. 4:**
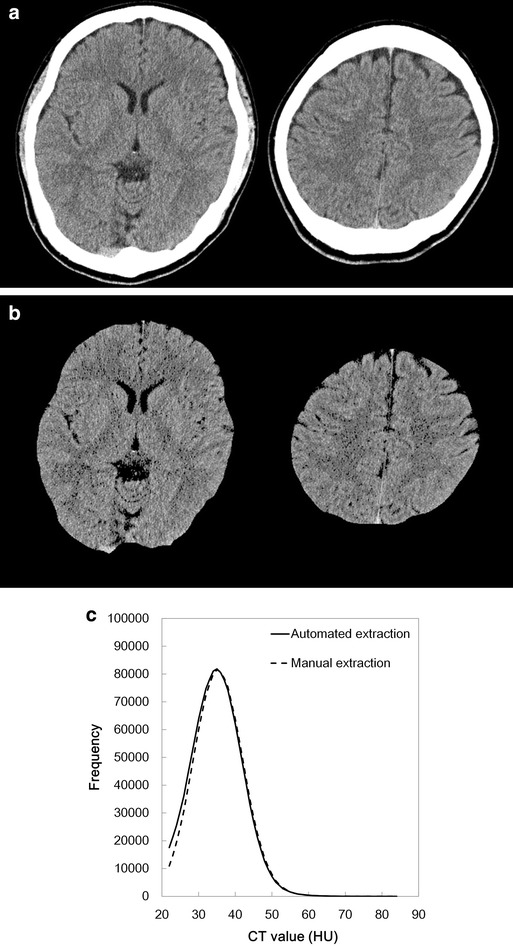
Representative CT images (**a**), extracted results from the corresponding slices (**b**), and CT histogram (**c**; *solid line*: automated extraction; *dashed line*: manual extraction) from a 27-year-old male. The similarity index was 0.937, which was the median of all the databases

**Fig. 5:**
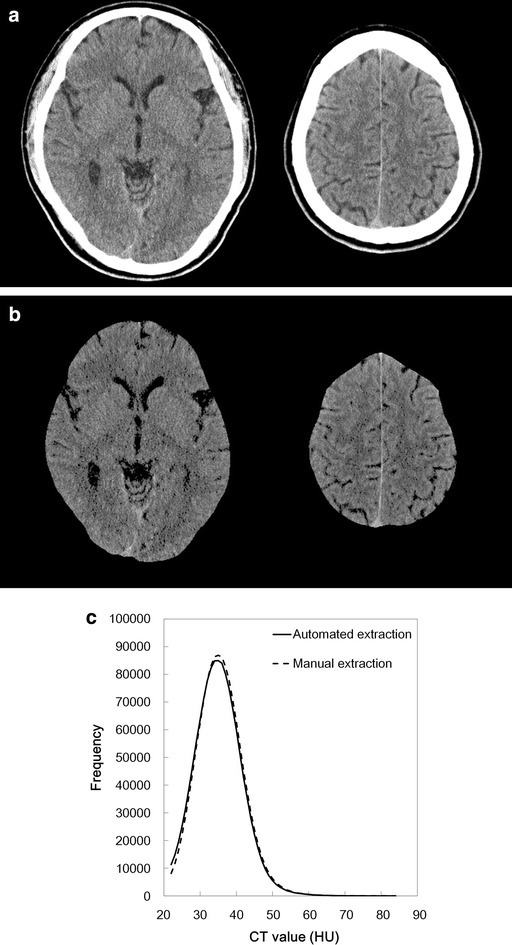
Representative CT images (**a**), extracted results from the corresponding slices (**b**), and CT histogram (**c**; *solid line*: automated extraction; *dashed line*: manual extraction) from a 16-year-old male. The similarity index was 0.908, which was the lowest of all the databases

The number of extracted pixels was higher with automated extraction (1,314,772 ± 95,705 than with manual extraction (1,280,377 ± 100,856; *p* = 0.010). The similarity index, false-positive rate, and false-negative rate were 0.93 ± 0.01, 8.3 ± 2.4 %, and 5.6 ± 0.6 %, respectively. Figures 3,  4, and 5 shows selected CT images and the extraction results from the subject with the highest similarity index (0.941), the median similarity index (0.937), and lowest similarity index (0.908), respectively.

### Histogram analysis

There was no significant difference in mean CT number, kurtosis, and skewness between automated extraction (35.0 ± 1.4 HU, 0.63 ± 0.07, and 0.51 ± 0.04, respectively) and manual extraction (35.4 ± 1.4 HU, 0.59 ± 0.07, and 0.46 ± 0.04 respectively; *p* = 0.5464, 0.7602, and 0.3936). There were excellent correlations between the automated and manual extractions for the mean CT number (r = 0.9743, *P* < 0.0001), kurtosis (r = 0.9892, *P* < 0.0001), and skewness (r = 0.9950, *P* < 0.0001). Fig 3c shows the histogram of CT numbers from the automated and manual extractions for the subject with the highest similarity index, and Fig. 4c shows the histogram with the middle similarity index, and Fig. 5c shows the histogram with the lowest similarity index.

## Discussion

In this study, we proposed an automated extraction method. It performed similarly to the manual extraction method for extracting the brain-tissue region from whole-brain CT images, as indicated by a high similarity index. Previous reports (Atkins and Mackiewich 1998; Shen et al. 2008) have stated that a similarity index >0.7 indicates excellent agreement with manual extraction on brain MR images. Our proposed automated extraction method achieved an excellent similarity index, well above this threshold. MR is often difficult to perform for physically or mentally unstable patients, and CT be may preferable, especially in emergency cases. We speculate that the few false positives and false negatives that did occur were caused mainly by partial-volume effect (Liu et al. 2003) of the cerebrospinal fluid around the brain. We therefore conclude that the accuracy of our automated extraction method is acceptable for clinical application.

Mean CT number, kurtosis, and skewness values are commonly used for quantifying histograms of CT number (Koyama et al. 2010; Best et al. 2003). Our present findings demonstrate that the mean CT number and kurtosis and skewness of the histogram of pixels identified by automated extraction were not significantly different from those of the pixels identified by manual extraction. In addition, we observed excellent correlations between the automated and manual extractions in all these histogram features (mean CT number, kurtosis, and skewness). These results support the histogram analysis of the brain-tissue region extracted from whole-brain CT images with the proposed automated extraction method is equivalent to that with the manual extraction.

We applied the fixed values for the thresholds in the automated extraction. CT value is influenced by the effective energy (Tanaka et al. 2006); therefore, the brain CT images by using the different scanning parameter or the different vender’s CT scanner might be influence to the performance of the proposal brain tissue extraction. The further study using brain CT images by using multi-scanning parameters from multi-CT scanners would demonstrate the more accurate result of the performance assessment of the automated extraction.

In conclusion, the proposal automated method well extracted the brain-tissue region from whole-brain CT images, and the histogram analysis of the brain-tissue region extracted from brain CT images with the proposed automated extraction method were equivalent to that with manual extraction.

## References

[CR1] Admiraal-Behloul F, van den Heuvel DM, Olofsen H, van Osch MJ, van der Grond J, van Buchem MA, Reiber JH (2005). Fully automatic segmentation of white matter hyperintensities in MR images of the elderly. Neuroimage.

[CR2] Atkins MS, Mackiewich BT (1998). Fully automatic segmentation of the brain in MRI. IEEE Trans. Med Imag.

[CR3] Bae KT, Fuangtharnthip P, Prasad SR, Joe BN, Heiken JP (2003). Adrenal masses: CT characterization with histogram analysis method. Radiology.

[CR4] Best AC, Lynch AM, Bozic CM, Miller D, Grunwald GK, Lynch DA (2003). Quantitative CT indexes in idiopathic pulmonary fibrosis: relationship with physiologic impairment. Radiology.

[CR5] Carmichael OT, Aizenstein HA, Davis SW, Becker JT, Thompson PM, Meltzer CC, Liu Y (2005). Atlas-based hippocampus segmentation in Alzheimer’s disease and mild cognitive impairment. Neuroimage.

[CR6] Chaudhry HS, Davenport MS, Nieman CM, Ho LM, Neville AM (2012). Histogram analysis of small solid renal masses: differentiating minimal fat angiomyolipoma from renal cell carcinoma. AJR.

[CR7] Cunningham FG, Twickler D (2000). Cerebral edema complicating eclampsia. Am J Obstet Gynecol.

[CR8] DeLeo JM, Schwartz M, Creasey H, Cutler N, Rapoport SI (1985). Computer-assisted categorization of brain computerized tomography pixels into cerebrospinal fluid, white matter, and gray matter. Comput Biomed Res.

[CR9] Dzialowski I, Weber J, Doerfler A, Forsting M, von Kummer R (2004). Brain tissue water uptake after middle cerebral artery occlusion assessed with CT. J Neuroimaging.

[CR10] Ganesan R, Radhakrishnan S (2009). Segmentation of computed tomography brain images using genetic algorithm. Int J Soft Comput.

[CR11] Gawler J, Du Boulay GH, Bull JW, Marshall J (1976). Computerised tomography (the EMI scanner): a comparison with pneumoence-phalography and ventriculography. J Neurol Psychiatry.

[CR12] Gould GA, Redpath AT, Ryan M, Warren PM, Best JJ, Flenley DC, MacNee W (1991). Lung CT density correlates with measurements of airflow limitation and the diffusing capacity. Eur Respir J.

[CR13] Gupta V, Ambrosius W, Qian G, Blazejewska A, Kazmierski R, Urbanik A, Nowinski WL (2010). Automatic segmentation of cerebrospinal fluid, white and gray matter in unenhanced computed tomography images. Acad Radiol.

[CR14] Hara T, Matoba N, Zhou X, Yokoi S, Aizawa H, Fujita H, Sakashita K, Matsuoka T (2007) Automated detection of extradural and subdural hematoma for contrast-enhanced CT images in emergency medical care. Proceedings of annual conference of SPIE medical imaging 651432

[CR15] Hu Q, Qian G, Aziz A, Nowinski KL (2005) Extraction of brain from computed tomography head images. In: Proceeding of 27th Annual conference of the Engineering in Medicine and Biology Society, p 3375–3378 10.1109/IEMBS.2005.1617201 17280946

[CR16] Kim EY, Ryoo JW, Roh HG, Lee KH, Kim SS, Song IC, Chang KH, Na DG (2004). Reversed discrepancy between CT and diffusion-weighted MR imaging in acute ischemic stroke. Am J Neuroradiol.

[CR17] Koyama H, Ohno Y, Yamazaki Y, Nogami M, Kusaka A, Murase K, Sugimura K (2010). Quantitatively assessed CT imaging measures of pulmonary interstitial pneumonia: effects of reconstruction algorithms on histogram parameters. Eur J Radiol.

[CR18] Liu J, Huang S, Ihar V, Ambrosius W, Lee LC, Nowinski WL (2003). Automatic model-guided segmentation of the human brain ventricular system from CT images. Acad Radiol.

[CR19] Maksimovic R, Stankovic S, Milovanovic D (2009). Computed tomography image analyzer: 3D reconstruction and segmentation applying active contour models—‘snakes’. Int J Med Inform.

[CR20] Sakai N, Mishima M, Nishimura K, Itoh H, Kuno K (1994). An automated method to assess the distribution of low attenuation areas on chest CT scans in chronic pulmonary emphysema patients. Chest.

[CR21] Shen S, Szameitat AJ, Sterr A (2008). Detection of infarct lesions from single MRI modality using inconsistency between voxel intensity and spatial location—a 3-D automatic approach. IEEE Trans Inf Technol Biomed.

[CR22] Soltanian-Zadeh H, Windham JP (1997). A multiresolution approach for contour extraction from brain images. Med Phys.

[CR23] Sugimori H, Kanna T, Yamashita K, Kuwashiro T, Yoshiura T, Zaitsu A, Hashizume M (2012). Early findings on brain computed tomography and the prognosis of post-cardiac arrest syndrome: application of the score for stroke patients. Resuscitation.

[CR24] Takahashi N, Satou C, Higuchi T, Shiotani M, Maeda H, Hirose Y (2010). Quantitative analysis of brain edema and swelling on early postmortem computed tomography: comparison with antemortem computed tomography. Jpn J Radiol.

[CR25] Tanaka C, Ueguchi T, Shimosegawa E, Sasaki N, Johkoh T, Nakamura H, Hatazawa J (2006). Effect of CT acquisition parameters in the detection of subtle hypoattenuation in acute cerebral infarction: a phantom study. Am J Neuroradiol.

[CR26] Yanagawa Y, Un-no Y, Sakamoto T, Okada Y (2005). Cerebral density on CT immediately after a successful resuscitation of cardiopulmonary arrest correlates with outcome. Resuscitation.

